# Process skill rather than motor skill seems to be a predictor of costs for rehabilitation after a stroke in working age; a longitudinal study with a 1 year follow up post discharge

**DOI:** 10.1186/1472-6963-7-209

**Published:** 2007-12-21

**Authors:** Ann Björkdahl, Katharina Stibrant Sunnerhagen

**Affiliations:** 1 Institute of Neuroscience and Physiology-Rehabilitation Medicine, Göteborg University, Sweden; 2 Arbetsterapin SU/Högsbo, B1, Box 301 10, S-400 43 Göteborg, Sweden

## Abstract

**Background:**

In recent years a number of costs of stroke studies have been conducted based on incidence or prevalence and estimating costs at a given time. As there still is a need for a deeper understanding of factors influencing these costs the aim of this study was to calculate the direct and indirect costs in a younger (<65) sample of stroke patients and to explore factors affecting the costs.

**Methods:**

Fifty-eight patients included in a study of home rehabilitation and followed for 1 year after discharge from the rehabilitation unit, were interviewed about their use of health care services, assistance, medications and assistive devices. Costs (defined as the cost for society) were calculated. A linear regression of cost and variables of functioning, ability, community integration and health-related quality of life was done.

**Results:**

Inpatient care contributed substantially to the direct cost with a mean length of stay of 92 days. Rehabilitation during the first year constituted of an average of 28 days in day clinics, 38 physiotherapy sessions and 20 occupational therapy sessions. The total direct mean cost was 80 020 € and the indirect cost 35 129 €. The direct costs were influenced by the process skill (the ability to plan and perform a given task and to adapt when needed) and presence of aphasia. Indirect costs for informal care giving increased for patients with a lower health-related quality of life as well as a low score on home integration.

**Conclusion:**

Costs are high in this group of young (< 65 years) stroke patients compared to other studies, partly due to the length of the stay and partly to loss of productivity.

## Background

Several studies have been done on the incidence/prevalence and cost of stroke [1-4] as well as on the long-term cost of illness in stroke [5-7]. The demand for studies of cost of stroke will continue to increase over the coming years as a result of the high prevalence of stroke and the frequent long-term consequences of survivors' disabilities, which represent a substantial socioeconomic burden associated with the disease. There is also a need for more detailed studies of data specific to the location of care and the resources consumed [5]. Payne *et al.*[5] also highlight the need for more studies that provide total cost estimates by stroke outcome, such as stroke severity. Three studies in the review mentioned above reported costs by age at the time of stroke and each showed a significant trend toward lower costs with increasing age (above 65 years of age) [8-10]. The authors suggest this to be due to the additional years of care for younger patients that result from longer survival after a stroke. However, greater rehabilitation efforts may also be made among these younger patients than among the elderly, as they have a need for a higher level of ability in their daily life, including work, child care etc.

As shown in the above studies, stroke is associated with high cost. However, the cost varies strongly depending on patient and system features [11] and comparisons of costs for stroke care are not as straightforward as for simpler interventions. Stroke is a complex disease that often occurs in people with multiple co-morbidities and the interventions are often not tested in randomized controlled trials [12]. There is a relation between age, lesion location and initial neurological deficit and functional outcome. Cost efficacy must be taken into consideration when priorities are set within the limited resources for health care. Most health economic studies done to date have focused on the cost efficacy of medical drugs or intervention; however, other aspects of stroke care, such as rehabilitation, must also be addressed.

In light of this, the aim of the present study was to describe the direct and indirect costs of hospitalization and rehabilitation in the first year after a stroke in "younger" persons (<65 years) and to examine the factors that contribute to higher costs. Based on prior studies we hypothesized that ability in activities of daily life and aphasia would influence costs.

## Methods

### Materials and methods

**Figure 1:**
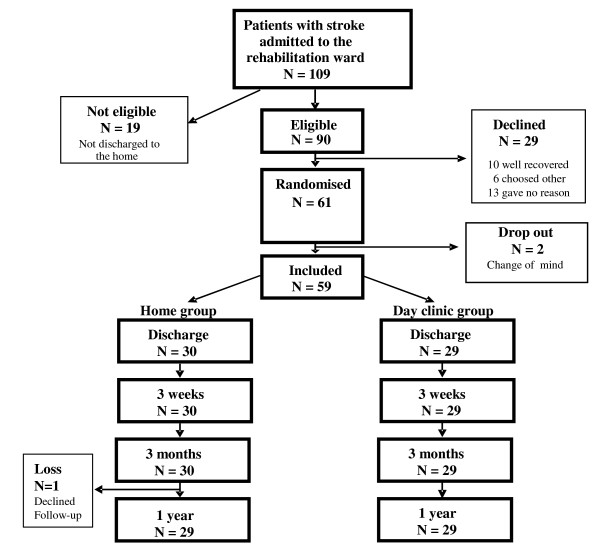
Flow chart of the eligible patients.

The paper focuses on patients with a first occurrence of stroke admitted to the rehabilitation clinic after the acute care at a stroke unit. The patients (< 65 years of age) were patients predicted to return to their home after a period of rehabilitation. Consecutively all patients with a first occurrence of stroke, discharged to there home (N = 90), were approached and 59 patients agreed to participate in the randomised controlled study, intending to assess the effects of three weeks of rehabilitation after discharge aiming at improved adaptation (Fig 1) [13]. Randomisation was performed the last week before discharge not to influence the length of stay. In this paper the analyses are based on data from 1 year follow-up and from both groups, N = 58.

### Costs

Costs are defined as the cost for society since the health and welfare systems in Sweden are tax financed. Hospitalization costs per hospital day were taken from estimates made by the civic administration of the city of Göteborg, differentiating between general ward, stroke unit and rehabilitation ward. The cost included both a "hotel" cost (staff costs, rent costs and overhead costs for food, medications, cleaning, washing and transportation) as well as a patient related cost for medical examinations and treatments. Estimated costs per day at the day clinic were obtained from the Sahlgrenska University Hospital economy department. The costs for other types of outpatient care were taken from estimates by the civic administration. The services recorded for the cost after discharge were visits to a physician, physiotherapist, occupational therapist, nurse, psychologist, speech therapist etc. e.g. the costs for the health care sector to supply the service. The patients were also asked about the amount of time they had home assistance, a personal assistant or aid from an informal caregiver. The costs for assistive devices, home modifications and medications were also recorded. These data were complemented with data from medical records concerning days in hospital after the stroke and readmittance during the first year after discharge. Detailed data about prescriptions and use of medications were obtained from the interview with the patient. The costs were then derived by taking the mean cost of the Swedish retail price of the different products of a substance and calculating the cost of the defined daily dosage multiplied by the number of days used during the follow-up year. The interview with the patients also provided information about what assistive devices the patient had received and the costs were calculated on the basis of Swedish retail prices. The costs for assistance were estimated by the civic administration as above and were differentiated between home assistance and personal assistance, e.g. the costs for society to supply the service.

The indirect cost consisted of two parts, production loss and assistance of informal caregiver. Production loss was estimated as average monthly salaries, divided according to men and women, including employment payroll taxes [14,15]. The other part of the indirect cost, assistance of informal caregiver, was the number of hours of informal care that were collected in the interview with the patients. The estimated cost per hour for an informal caregiver was taken from the work of Claesson *et al.*[4], from the same university. All costs are calculated according to 2004 prices in Swedish crowns (SEK, exchange rate 2004, 1 € = 9.22 SEK).

### Instruments

Data was gathered at discharge, three weeks, three months and one year post discharge by persons not involved in the intervention (blinded).

The Assessment of Motor and Process Skills (AMPS) is an observational measure used to measure the quality of performance of instrumental activities of daily living (IADL) tasks. The AMPS evaluates two domains of occupational performance, i.e. ADL motor and process skills in 16 ADL motor skill items and 20 ADL process skill items. Motor skills are defined as the observable goal directed actions the persons enacts during the performance of ADL tasks in order to move oneself or the task objects. Process skills are defined as the observable actions of performance the persons execute to logically sequence the actions of the ADL task performance over time, select and use appropriate equipment and adapt his or her performance when problems are encountered. The occupational therapist observes the subject perform two ADL tasks and scores the performance in each item on a four-point scale, where 4 = competent, 3 = questionable, 2 = ineffective and 1 = markedly deficient. The raw scores are then entered into the AMPS computer program which converts the ordinal data into a linear measure (logit) of ability in motor and process skills [16,17]. Data from discharge was used.

The National Institute of Health Stroke Scale (NIHSS) [18] is a quantitative measure of neurological deficits. The items are summarized (maximum 36) and a lower score indicates fewer deficits [18]. Presence of aphasia was decided according to the NIHSS at discharge.

Euroqol, EQ-5D, is a generic instrument for measurements of health related quality of life (HRQoL) [19]. The EQ-5D includes a visual analogue scale on which the patients rate their own health between 0 and 100. The data used in this study are taken from the visual analogue scale at one year post discharge.

The Community Integration Questionnaire (CIQ) is a 15-item scale that provides a total score for the extent of community integration (higher scores show greater integration) and subscale scores for home integration, social integration and productive activity [20]. Data was used from one year post discharge.

Functional Independence Measure consists of 13 motor (physical) items and 5 social-cognitive items [21], assessing dependence with ratings from 1 as totally dependent to 7 as independent [22]. Functional Independence Measure has been validated [23,24] and examined for use in Sweden [25]. Discharge data was used.

In the 30 metres walking test the person is requested to walk indoors at his/her own speed and the velocity is recorded (m/s) [26]. Data from discharge was used.

### Data analysis

Mean and median costs for hospital days, the different rehabilitation services, assistance, assistive devices, home modifications and medicine were calculated, as was the total direct cost. The indirect cost was divided into cost of assistance provided by informal caregivers and production loss and is also given as a total indirect cost.

A linear regression was done to examine the factors contributing to higher costs (SPSS 11.0 with the method, enter). The selection of variables for the regression was made by generating a hypothesis of relevant factors that might affect the cost based on scientific findings concerning the consequences of a stroke. The hypothesis was that activity level, ability to walk and presence of aphasia were factors that might possibly influence the direct cost, i.e. length of stay in hospital (LOS) and need for rehabilitation services and aids. The activity level was represented by the two ability measures, motor and process skill, on the Assessment of Motor and Process Skill scale (AMPS). Walking ability was recorded with a 30-metre walking test given in m/s, and aphasia was given as three categories: no aphasia, mild and severe aphasia assessed by the National Institute of Health stroke scale (NIHSS).

The hypothesis for the indirect costs of assistance from informal caregivers was that they might be influenced by the stroke victim's perceived HRQoL (EQ-5D), activity level, presence of aphasia and his/her participation in daily activities in the home (CIQ). In this study, we used the CIQ subscale of home integration, which was defined as: 0–3 = not integrated and >3–10 = integrated.

Multicollinearity was checked for and not found to be of concern.

## Results

The sample of stroke patients was relatively young, with a median age of 53 (Table 1), and most of them were, as far as they knew, quite healthy prior to the stroke. However, 80% of the patients (47/58) had co-morbidities that affected their rehabilitation after the stroke. The most common co-morbidities in this sample were hypertension (32% of the patients), depression (25%), diabetes (17%), heart failure (8%) and alcohol abuse (7%). Two patients were blind and one had a severe hearing deficit. Before the stroke, 3 of the 58 patients were on early retirement, and the others either worked (52) or were actively looking for work (3). At the one-year follow-up only four of the patients had returned to work.

**Table 1:** Description of the sample

			% of the group	% in Sweden
Age in years	Median (range)	53 (27–64)		
	Mean (SD)	52 (7,67)		
Gender (number of patients)	Men	44	76%	65%
	Women	14	24%	35%
Type of lesion (number of patients)	Haemorrhage	17	29%	≅ 22%
	Cerebellar haemorrhage	3	5%	
	Cerebral infarction	36	62%	≅ 78%
	Cerebellar infarction	2	3%	
Location (number of patients)	Left hemisphere lesion	28	48%	
	Right hemisphere lesion	26	45%	
	Bilateral lesion	4	7%	
Living situation (number of patients)	Single	26	45%	
	Cohabitant	32	55%	
Type of ward (number of patients)	Stroke unit	37	64%	70%
	Other	21	36%	30%
Days as in-patient	Acute care, median (range)	25,5 (7–70)		
	Acute care, mean (SD)	28,8 (14,0)		
	Rehabilitation, median (range)	58 (20–155)		
	Rehabilitation, mean (SD)	63,5 (29,8)		

The sample of stroke patients seems to be representative for this age group in Sweden [27] (Table 1). The remaining impact of the stroke at discharge was quite low, with a median of 5 on the National Institute of Health Stroke Scale (the lower score the better) and with a median of 78 on the Functional Independence Measure motor sum score (total independence 91). According to the five severity levels defined by Caro *et al.*[28] on the basis of NIHSS, this sample consisted of patients with a very mild (NIHSS 0–9) or mild stroke (NIHSS 10–12) at discharge.

In Table 2 the different costs are shown. The mean time in hospital (acute care and rehabilitation) due to the stroke was 92 days (36–189) to a mean cost of 46 446 €. During the first year after discharge the patients received a great deal of rehabilitation. The mean number of visits to the day clinic the first year after discharge (the first three weeks of intervention in the study not included) was 28 days to a mean cost of 13 802 €. Many also received additional training by a physiotherapist and occupational therapist during the year, with a mean number of visits of 38 and 20, respectively. The direct cost for the first year per patient, including different kinds of rehabilitation services, home assistance, assistive devices and medication, was 33 604 € (2256 – 137 133). During the first year after discharge, 18 of the patients (31%) had been admitted to the hospital at an average cost of 2076 €. The length of stay varied between 1–21 days, median 1 day. One person suffered a second stroke (20 days), 2 suffered from debut of epilepsy, and one required hospitalization due to severe depression (21 days), the rest had a mix of reasons such as stomach cramps, fractures etc which required 1–2 days of observation or interventions. The mean indirect cost per patient including production loss and cost of aid from informal caregivers was 35129 €. In total, an average direct and indirect cost for a stroke patient including cost for hospitalization after the stroke and costs during the first year after discharge was 115 179 €.

**Table 2:** Use of resources and cost for hospitalization after stroke and in the first year after discharge.

			**Number**			**Cost in €**		
			Mean	Median	Min-Max	Mean	Median	Min-Max

Direct cost	Acute care in hospital	days	29	25,5	7 – 70	11 401	10 085	2768 – 27 684
	Rehabilitation ward	days	63	58	20 – 155	35 046	32 019	11035 – 85 567
	Day clinic rehabilitation	days	28	24	0 – 87	13 802	11 471	0 – 41 581
	Visit to physician	number	4	3	0 – 30	1 301	977	0 – 9 765
	Visit to physiotherapist	number	38	28	0 – 169	3 113	2 253	0 – 13 844
	Visit to occupational therapist	number	20	6	0 – 169	1 679	573	0 – 13 844
	Visit to nurse	number	16	6	0 – 183	788	283	0 – 8 637
	Home assistance	hour/week	0.64	0	0 – 8	1 017	0	0 – 11 645
	Personal assistant	hour/week	4.77	0	0 – 120	5 243	0	0 – 98 679
	Assistive devices	number	3.19	2	0 – 13	442	173	0 – 4 662
	Transportation service for disabled	trips/week	3.65	2.5	0 – 36	3 413	2 344	0 – 27 227
	Housing adaptations				935	0	0 – 10 850
	Medication				593	451	0 – 4 126
Indirect cost	Informal caregiver	hour/week	15.12	2	0 – 63	2 677	1 312	0 – 10 663
	Production loss first year				29 452	33 592	0 – 33 592

Summary	Inpatient care	days	92	89.50	36 – 189	46 446	44 921	17 687 – 986 148
	Direct cost first year after discharge				33 604	31 353	2 256 – 137 133
	Total indirect cost first year after discharge				35 129	34 904	0 – 44 255
Total	Inpatient care, direct and indirect cost one year after discharge				115 179	111 178	

In the regression analysis of direct costs the determinants "severe aphasia" and "process skill" (AMPS) were statistically significant. With one logit higher process skill, the cost decreased by 16 920 € (156 098 SEK) and the cost for a patient without aphasia was 34 165 € (314 928 SEK) less than a patient with severe aphasia (Table 3).

**Table 3:** Linear regression of direct costs and factors affecting the cost.

**Direct costs**		**Model**	Parameter estimates	Std. Error	Sign.
Adjusted R^2^	0.373				
Sign.	0.000				
		Intercept	1027 757	99 650	0.000
		Mild aphasia	22 860	118 075	0.847
		Severe aphasia	314 928	111 617	0.007
		Walking	-217 887	126 288	0.092
		Motor ability (AMPS)	-4 446	66 068	0.947
		Process ability (AMPS)	-156 098	74 289	0.042

In the regression analysis of the cost for assistance of informal care giving the determinants "home integration" and HRQol were statistically significant. Compared to not being integrated in the home, an integrated patient cost 3 623 € (33383 SEK) less in terms of costs for informal care giving. For each degree higher on the EQ-5D thermometer from 0–100, the cost for informal care giving was almost 65 € (572 SEK) less (Table 4).

**Table 4:** Linear regression of indirect costs and factors affecting the cost.

**Indirect costs**		**Model**	Parameter estimates	Std. Error	Sign.
Adjusted R^2^	0.484				
Sign.	0.003				
		Intercept	60 719	21 389	0.010
		Mild aphasia	6 133	14 781	0.682
		Severe aphasia	-7 254	14 077	0.612
		Walking	13 339	18 700	0.484
		Motor ability (AMPS)	-5 880	7 246	0.426
		Process ability (AMPS)	-7 056	10 440	0.507
		HRQol (EQ-5D)	-572	244	0.029
		Home integration(CIQ)	33 383	10 282	0.004

## Discussion

Extensive resources are used for hospitalization and rehabilitation the first year after a stroke. The sample of patients in this study is representative for persons with stroke in this age in Sweden, with a "high" proportion of men and also of haemorrhage as cause of stroke [27]. In this sample of "younger" patients (< 65) the indirect costs of production loss are also significant as most patients were productive at onset and only four had resumed work one year after discharge. The direct costs after a stroke are significantly influenced by the process skill, i.e. the ability to plan and perform a given task and to adapt when needed. The other factor found to influence the cost is the presence of aphasia. The informal caregiver makes a substantial assistance contribution when the patient is not able to participate in home duties and he or she perceives low health related quality of life.

The total direct cost in the present study consisted of costs for acute hospitalization and costs for rehabilitation and care associated with the stroke during the first year after discharge. The cost for hospitalization made the largest contribution to the total cost, almost 60%. Patients conventionally receive a substantial part of their rehabilitation in hospital, for which reason different services have been developed to reduce the length of stay (LOS) such as Early Supported Discharge, ESD. The costs and effect of ESD services have been studied in several trials [29] showing that ESD services provided for a selected group of elderly stroke patients can reduce the length of hospital stays. However, more research is required to define the important characteristics of effective ESD services and to define the balance of cost and benefit for different patients and service groups. This suggests a need to establish whether there are differences between different age groups. In the present study of "younger" (<65) patients the mean LOS in hospital after the stroke was 92 days (acute care 29 days), compared to the study by Claesson *et al.*[4] of elderly stroke patients (age >70) at the same hospital, where the mean LOS was 28 days (acute care 11 days). The average LOS after a first stroke event in Sweden is 28 days [2]. In Sweden, the official policy is that age should not be a factor in setting priorities, but rather only the need for health care. There is a wide variation in the literature on the relationship between age and LOS. However, Black-Schaffer and Winston [30] found an association between age (the young groups, <55, 55–64 and 3 older groups) and LOS, where LOS shortened with each successive age group, even though the LOS efficiency, i.e. gains in FIM points per day, had a significant relationship with younger age. This seems counterintuitive as older patients should then need longer inpatient rehabilitation if the LOS efficiency is lower with higher age. LOS, however, is sensitive to a variety of non-medical factors, including team culture, which may set higher goals for younger patients.

Evers *et al.*[31] suggest a model of three factors that contribute to the volume of hospital utilization divided into predisposing factors, enabling factors and need factors. Predisposing factors refer to service use related to individual background characteristics, such as functional level before stroke and previous periods of illness. An explanation for the difference in LOS between age groups with respect to this factor might be that the younger patients do not have a previous history of co-morbidities and thereby need a more extensive investigation after the stroke. Lee *et al.*[32] explain the shorter LOS among elderly patients with the use of a less aggressive approach. Enabling factors are those which make health services resources available to the patient. In a Dutch study, 36% of the total LOS among stroke patients was explained by non-medical reasons [33]. For the elderly patient with a long medical history, placement in a nursing home or home assistance may already exist. In this younger sample, all returned to their own homes and in most situations expected to be alone during the day, which could contribute to a longer stay in hospital. Need factors refer to the current level of illness in patients including clinical complications and co-morbidities. In most studies of the cost of stroke, this single factor is used to explain the cost. The sample in the present study consisted mostly of stroke patients with a moderate stroke at admission, and 80% had co-morbidities that affected the rehabilitation. Patients who die during the hospital stay after a stroke generally tend to have shorter stays, thereby reducing costs. The sample in the present study was recruited from the rehabilitation ward and the most critical period had thus elapsed.

The resources used for outpatient rehabilitation also differ significantly between this study and Claesson *et al.*[4]. The elderly patients received an average of less than three days per patient of outpatient rehabilitation from discharge to 12 months, as compared to 28 days in the younger group. After the period of natural recovery the issues that play a part in stroke outcome are related to the return to normal activities and overall quality of life [34]. The impact on the lives of the individuals affected by stroke can vary depending on the disability and the demands placed by normal activities. For people in working age, it automatically means that normal activities for most include employment and active involvement in the family. Gainful employment is often a rationale for rehabilitation, since employment results in financial independence [35]. Levy *et al.*[36] compared stroke care costs in Europe and noted small differences in acute stroke care costs but large variations in rehabilitation costs resulting in a ten-fold variation in follow-up care. The same variation may also be present in different groups of patients, such as different age groups. In the comparison of inpatient care between age groups in the present study and elderly patients in the same city, the greatest differences were found in the use of resources for rehabilitation and follow-up.

In examining the factors that significantly contributed to higher direct costs of the acute hospitalization and rehabilitation during the first year after discharge we found that a patient with severe aphasia costs 34 165 € (314 928 SEK) more than a patient with no aphasia, and each logit of lower ability on the AMPS process skill scale increases the cost by 16 920 € (156 098 SEK). LOS can be related to level of impairment [37], which seems also to be the case in this study. Three patients had somewhat higher costs than the rest and of these; two had severe aphasia and severe physical disability, and one severe physical disability and cognitive deficit.

The findings in the present study suggest that process skills are of importance; however this has not received much attention in prior studies or in clinical work. Cognitive deficits following stroke, which strongly influence the process skill, are being increasingly recognized as an important factor in determining return to work and the ability to resume normal activities [38]. The findings that women cost less after stroke since they receive less extensive examination and treatment [39,40] can neither be confirmed nor refuted in this sample as the number of patients was too small.

Indirect costs for assistance from informal caregivers in the present study were found to be related to the patients' health related quality of life and home integration. Functional disability and mood disorder may independently contribute to the restricted participation of post-stroke patients [41]. When estimating the need for different health services the most common approach is to use instruments that measure function or ability that can explain that the direct costs are related to these aspects. To the contrary, the need of assistance seems to originate from other factors related more to the ICF term participation [42]. D'Alisa *et al.*[41] found depression to be a determinant factor for social integration. Participation in the home is an important context in which the individual develops positive life satisfaction [35]. The role of the informal caregiver could not only be to take care of things in the home that would not otherwise be done, but also to help the patient to participate.

A limitation in this study is the relatively small sample, which makes it impossible to divide the patients into subgroups and might make generalization more difficult. However, the small sample allows a greater possibility to gather detailed information. As the sample is small and hence the power of the regression is low (power calculations made after the regression analysis ranged between 0.06 and 0.80) the interpretation of the non significant results must be done with care. Non significant results does not exclude that those variables could be of importance. However, the significant variables found contribute with important information about what could affect cost even though there may be additional variables of importance.

The group examined is part of the younger stroke population – the 20% of the stroke population in Sweden who are in working age – and the situation after stroke in this group is different from that of the average stroke person aged 75 [27]. The sample consisted of patients in the Swedish health care system, referred to the rehabilitation clinic, which implies patients with a moderate to severe stroke, and costs may vary with stroke severity and health care systems.

## Conclusion

Process skill rather than motor skill is a significant predictor of costs for rehabilitation of a stroke patient in working age and may be given more attention. The presence of aphasia is another factor that increases the cost. Costs are high in this selected group of "younger" patients with stroke compared to other studies of stroke. This is in part due to length of stay and in part to loss of productivity.

## Competing interests

The author(s) declare that they have no competing interests.

## Authors' contributions

AB has taken part in the design of the study, gathered most of the data, performed the analysis and done most of the writing. KS has gathered some of the data and participated in analysis and in the writing. All authors have read and approved the final manuscript.

## Pre-publication history

The pre-publication history for this paper can be accessed here:


